# Study protocol for a randomized controlled trial to adapt a posttraumatic stress disorder intervention of patients with opioid-stimulant polysubstance use receiving methadone maintenance treatment

**DOI:** 10.1186/s12888-024-06348-0

**Published:** 2024-12-04

**Authors:** Tanya Renn, Brittany Griffin, Vinodini Kumaravelu, Ana Ventuneac, Michele Santacatterina, Amanda M. Bunting

**Affiliations:** 1 College of Social Work, Florida State University, Tallahassee, FL USA; 2 StartCare, Brooklyn, NY USA; 3 Department of Population Health, New York University Grossman School of Medicine, New York, NY USA

**Keywords:** Post-traumatic stress disorder (PTSD), Methadone-maintenance treatment (MMT), Polysubstance use, Opioid, Cocaine, Trauma informed care

## Abstract

**Background:**

The purpose of the Treatment for Harnessing Resiliency, Improving emotional regulation, and empowering indiViduals for a brighter future (THRIVE) study is to adapt an evidence-based posttraumatic stress disorder (PTSD) treatment for use among a polysubstance population receiving methadone maintenance treatment (MMT) at an opioid treatment program. Polysubstance use of high-risk combinations, such as illicit opioids and stimulants, is a critical public health issue. Individuals who engage in these high-risk combinations are more likely to have histories of childhood trauma, multiple traumas, PTSD, and greater PTSD severity as compared to mono-substance using individuals. Trauma, co-morbid mental health disorders such as PTSD, and polysubstance use complicate treatment outcomes. This study will use eight study phases to adapt an existing evidence-based PTSD intervention, Skills Training in Affective and Interpersonal Regulation with Narrative Therapy (STAIR-NT), via a massed treatment model (i.e., condensed treatment schedule) for patients in MMT who are engaged in sustained opioid-stimulant polysubstance use.

**Methods and analysis:**

The intervention is an adapted version of the STAIR-NT protocol. The massed version created includes four 60-min sessions of skill building and two weeks of four 60-min sessions of narrative therapy. A preliminary randomized controlled trial (RCT) with 80 participants, randomized 1:1, will be conducted to assess the intervention's implementation and impact on primary short-term outcomes of polysubstance use and PTSD symptoms.

**Ethics and dissemination:**

The results of this study will inform a fully-powered effectiveness trial for individuals with PTSD and polysubstance use receiving MMT. The findings are expected to provide valuable insights into improving both PTSD and substance use outcomes, and real-world implementation insights to integrating trauma-informed care in treatment settings for vulnerable populations.

**Registration:**

This study is registered at ClinicalTrials.Gov as NCT06307340. Registration date 03/2024.

## Background

Polysubstance use (PSU) is a critical public health issue. Evidence indicates that the majority of persons who use drugs engage in PSU; a pattern of diverse drug involvement that may include substances used concurrently (i.e., substances used on separate occasions) or simultaneously (i.e., two or more substances used in a single occasion) [1–5]. The co-use of opioids with stimulants substantially contributes to overdose fatalities. In 2022 alone, an estimated 1.5 million years of life were lost due to the co-use of opioids and stimulants [6]. Notably, cocaine deaths with co-occurring opioid PSU are growing at a faster rate than overdoses involving only cocaine, and the Northeastern United States faces a significantly disproportionate burden of cocaine and opioid co-involved overdoses [7, 8]. Further, rates of cocaine-opioid overdose fatalities are growing faster among Black Americans, revealing deepening inequities within the current polysubstance health crisis [9]. PSU is particularly pronounced among vulnerable subpopulations such as individuals with severe substance use disorders and those with histories of trauma [10–15].

Evidence suggests that PSU complicates treatment outcomes [16–20]. This effect may be particularly acute for individuals receiving medication for opioid use disorder including those receiving methadone maintenance treatment (MMT). An estimated 30–70% of patients in MMT use cocaine [21–26]. While cocaine use may decrease during MMT for some patients [27, 28], there is a subset of patients (as many as 40%) who engage in chronic and persistent use of cocaine during MMT [25, 26, 28–31]. These individuals are twice as likely to also sustain their use of opioids [25, 32]. The continued use of cocaine and other opioids is associated with poor MMT retention [21, 33, 34] as well as persistent injection drug use behaviors [21, 35], increasing the risk of injection harms and overdose.

The majority of individuals in MMT have trauma histories, and the prevalence of posttraumatic stress disorder (PTSD) is estimated at 30% [36]. However, this number is thought to be undercounted due to a lack of utilization of clinical screening tools [37]. The ongoing use of stimulants and other drugs during MMT may be particularly acute for individuals with trauma histories [15, 37, 38]. Individuals who engage in PSU have increased incidence of PTSD, have more severe PTSD symptomatology, and an increased incidence of childhood trauma [11–14, 39]. However, PTSD often goes unrecognized and untreated in SUD treatment settings [40–42]. Left unaddressed, PTSD symptoms can worsen following treatment for SUD [41, 43]. Concurrent treatment of PTSD and substance use can improve treatment retention and overall psychological wellbeing for patients [44]. Addressing PTSD among individuals engaged in MMT represents a critical opportunity to provide effective treatment that is responsive to the needs of individuals who engage in sustained opioid-stimulant PSU, thereby reducing the severity of PSU and attendant risks of overdose.

### Treatment of PTSD in substance use treatment settings

Several promising approaches to treating PTSD and substance use are emerging [45] yet no standard exists for treating this co-morbid population. Further, different approaches may be warranted for different SUD settings given the heterogeneity of treatment settings and differing organizational strengths and limitations.

The current study utilizes an adapted version of the Skills Training in Affective and Interpersonal Regulation with Narrative Therapy (STAIR-NT) intervention. STAIR-NT is an established three-module program across 18 sessions [46, 47]. In traditional delivery, the first module consists of five sessions dedicated to the development of core skills related to emotional competencies and the second five session module is dedicated to the development of social competencies. Together, the modules create the STAIR portion of the intervention which provides individuals with the skills that trauma has stunted. The Narrative Therapy (NT) component is the third module when individuals confront their traumatic experiences. The emotional processing of traumatic memories becomes possible with improved self-regulation capacities and self-compassion provided during the STAIR sessions. Providing emotional regulation and skills training prior to NT improved treatment drop-out and PTSD symptomology in head-to-head comparison studies of the original treatment comparing STAIR-NT: STAIR + Support (no narrative therapy): exposure therapy (no STAIR) [48]. Previously, a flexible real-world approach was used to integrate STAIR-NT into community mental health settings, and individuals experienced improved emotional regulation and reduced use of substances to cope [49]. The intervention can be delivered in-person or via a web-based platform and has many opportunities for adaptions including a massed (i.e., condensed) treatment schedule.

Massed treatment models overcome limitations of traditional PTSD treatments by condensing evidence-based treatments into shorter timeframes [50]. In massed treatment, evidence-based trauma interventions that have typically been delivered in 12 to 18-week formats are condensed into periods ranging from 7 days to 3 weeks with comparable treatment outcomes [51]. A massed treatment model for PTSD may be particularly feasible and efficacious in opioid treatment settings, as prolonged models can compete against other clinical demands and increase the risk of treatment drop-out. An adaption of STAIR for primary care has been condensed into a 5-session version with demonstrated efficacy [52], and the demonstrated efficacy of that RCT indicates that the number of sessions and content can be adapted for other venues and to meet client needs [46].

### Study overview

The **T**reatment for Harnessing Resiliency, Improving emotional regulation, and empowering indiViduals for a brighter future (THRIVE) study aims to establish feasibility, acceptability, and appropriateness of the adapted STAIR-NT intervention as well as preliminary data on short-term outcomes of PSU and PTSD symptomology. This study consists of an adaption process using the ADAPT-ITT framework, provisional protocol testing in an open pilot, and an RCT. Briefly, the adaption process included qualitative feedback from key stakeholders (i.e., clinicians, community-based agency leaders, target PSU population), topical expert review, and an iterative process of protocol adaptations. After the completion of the provisional protocol, an open pilot was used to test and further refine the protocol.

The focus of this protocol are the study procedures for the RCT. Participants are randomized 1:1 to receive the adapted STAIR-NT or treatment as usual (TAU). The study is conducted in collaboration with the community partner site and opioid treatment program (OTP), StartCare (formerly START Treatment & Recovery Inc). The study was peer-reviewed as part of the funding selection process by the National Institutes of Health, USA.

## Methods

### Human ethics and consent to participate

The research protocol has been approved by the New York University Langone Health Institutional Review Board (IRB) and the START IRB. This study will be conducted in accordance with the Code of Federal Regulations on the Protection of Human Subjects (45 CFR Part 46). All participants will provide written consent to participate. A Certificate of Confidentiality is automatically obtained from the NIH. This study was registered 03/2024 at ClinicalTrials.Gov as NCT06307340.

### Study setting

StartCare is a community-focused, Black-founded, and BIPOC-operated organization with seven OTPs in New York City providing treatment services to more than 53,000 people with opioid use disorders since 1969. In 2023, StartCare provided care to over 3,300 patients, 95% of whom had an income below the federal poverty level and had Medicaid (76%), Medicare (10%), or both (13%) as their health coverage. The majority of patients were 55 years of age or older (54%), male (71%), and African-American, Hispanic, or mixed/other (84%), and 80% had been receiving services at StartCare for 12 months or longer. The behavioral health team, trained in motivational interviewing and cognitive behavioral therapy approaches as well as in delivering psychoeducational therapy and other evidence-based behavioral interventions, consists of peer advocates, certified alcohol and substance use counselors, licensed master social workers (LMSW) or licensed mental health counselors (LMHC), psychiatrists and psychiatric nurse practitioners, and behavioral health practitioners. One OTP site will be utilized for the RCT, and an LMSW and/or LMHC will be selected to serve as study interventionist(s).

### Sample

A total of 80 participants will be enrolled over 9-months. Participants must meet the following inclusion criteria: (a) be 18 years or older, (b) be a patient at the StartCare clinic receiving methadone for the treatment of opioid use disorder, (c) self-report 10 + days of co-use of cocaine and illicit opioids in the past 30-days, (d) meet the criteria for stimulant use disorder (cocaine type; mild, moderate or severe) and (e) screen for PTSD (score of ≥ 3 on the PC-PTSD-5) [53, 54]. Reported PSU can include both concurrent (i.e., substances used on separate occasions in the 30 days) and simultaneous (i.e., two or more substances used in a single occasion) use. Self-reported PSU will be measured using the drug use section of the Addiction Severity Index (ASI) [55]. Participants must meet the *Diagnostic and Statistical Manual of Mental Disorders, Fifth Edition (DSM-V)* [56] criteria for stimulant use disorder with at least mild severity (2 + symptoms). PTSD will be assessed via the PC-PTSD screener for DSM-V [53]. This screener first has a dichotomous question about lifetime trauma exposure. If endorsed negatively, the PC-PTSD-5 is complete with a score of 0. If endorsed positively, individuals then answer five brief dichotomous (yes/no) questions regarding disruption to their life in the past month. A score of 3 is selected as the cut-off based on the previous validation study [54].

Exclusion criteria include (a) cognitive impairment that would interfere with their ability to understand study participation as assessed by the researcher, (b) does not speak/understand English at a conversational level, (c) plans to leave the StartCare clinic in the next 60 days, (d) patients who missed methadone doses (inactive) for 30 days or more, or (e) having received clinical care from the interventionist(s) in the past 30 days.

### Sample size

Power calculations for the primary short-term outcome of change in number of days of PSU were conducted. To estimate the variability of number of days of PSU the manuscript of Kelly et. al [57] was used which studies a population of subjects in MMT, a decent equivalent of the proposed TAU intervention. The authors [57] reported a standard deviation of 6.78 and 7.85 days of heroin and cocaine use, respectively. The mean difference of PSU between arms was then assumed to be 5, leading to a medium-large effect size of around 0.7 (5 divided by the average of 6.78 and 7.85 days). With a sample of 30 participants per arm, the current proposed study will achieve 80% power to detect a size of 0.7. To account for a possible attrition rate of 15–20% of participants, the sample size was inflated while maintaining 80% power to 40 participants per arm.

### Randomization

Participants who screen eligible and consent will be randomized 1:1 to the adapted STAIR-NT intervention or TAU using randomization blocks of two and four via a computer-generated randomization sequence. Blinding of staff is not possible given the need to coordinate and deliver the intervention, however, the data analyst will be masked as to the group allocation.

### Intervention description

Based on study adaption processes, the intervention protocol is a massed version of STAR-NT to deliver eight 60-min sessions across six weeks. Sessions include content from the STAIR-NT intervention manual [46]. The first four sessions include STAIR components focused on emotional regulation (two sessions) and social competencies (two sessions). Then NT occurs for two-sessions per week over a two-week period processing a single traumatic/salient memory. Emotional competency topics in sessions one and two include psychoeducation of the impact of trauma on emotions and relationships, emotional awareness, and emotional regulation. Specific skills for emotional regulation that participants learn include skills in each channel of emotion (thoughts, body, behavior). Participants practice these skills in session with the interventionist. Additionally, participants complete interactive worksheets that assist them in identifying their emotions, understanding the context that brought on those emotions, and regulation behaviors to reduce emotional intensity. Social competency skills taught in sessions three and four include understanding the differences between assertiveness and aggressiveness, reviewing the impact of trauma on relationships, and understanding power dynamics. Participants conduct a role play with the interventionist to review current relationship patterns and revise their role play to incorporate the skills they learned. Additionally, participants complete an interactive worksheet with the interventionist regarding their current relationship patterns. Each session participants are provided with worksheets to take home with details about each topic and handouts to facilitate continued practice.

The NT component of the therapy begins in session five, when participants identify the traumatic memory they would like to address. The NT is audio recorded, and in first person, present language the participant discusses the traumatic memory. The interventionist encourages the participant to provide more details where appropriate. The participant and interventionist listen to the audio recording together to explore beliefs about self and others in the narrative, and continue to repeat this process− making new recordings, re−listening− over the remainder of the NT sessions (4 sessions in total).

### Treatment as usual (TAU)

Participants assigned to TAU will not have any additional components added to their MMT by the study. Participants will continue their MMT plan in accordance with their counselor at StartCare.

### Fidelity monitoring

Interventionists were trained by project consultant and lead trainer of STAIR institute. Interventionists complete session checklists for each participant to indicate the adherence to protocol and measure participant’s receipt of content. Additionally, weekly group supervision meetings will be recorded, transcribed, and qualitatively analyzed to examine barriers to fidelity.

## Procedures

### Recruitment

All participants will be recruited through the participating community partner, StartCare. Both pre-screening and screening tactics will be used. A trained research assistant will utilize StartCare treatment data on toxicology for the last three months and prioritize in-clinic contact with those individuals. Additionally, passive referral methods (i.e., IRB-approved flyers) and clinician referral will be used for recruitment. The research assistant is in-clinic with participants and most recruitment will occur in-person. Interested individuals may provide contact information and receive a follow-up phone call from research staff to complete the screening process. Recruitment is expected to be completed by June 2025.

### Randomization procedure

After completion of baseline, participants are randomized to the intervention or TAU. Randomization is programmed into REDCap [58, 59] by the biostatistician. All other research members are blinded to the order of block size and will not have access to the randomization schedule.

### Assessments

Participants complete a baseline and three follow−up waves of assessment at 6−weeks, 3−, and 6−month post−baseline. Assessment windows are −2 weeks/+ 6 weeks, and the 6−week assessment is timed to the end of the intervention for participants in that arm. Study assessments will be conducted by trained research staff. Assessments will primarily be completed by StartCare research staff, with the availability and allowances of NYULH research staff to engage with those who would prefer follow−up at a NYULH location, or those no longer engaged with the StartCare clinic. Follow−up assessments will repeat relevant baseline measures, with intervention participants receiving a brief measure of intervention satisfaction at their first follow−up (see Table 1). To increase data harmonization, the use of common data elements and PhenX Toolkit measures are proposed [60]. Assessments will be conducted via Computer−Assisted Personal Interviews (CAPI) using the NYULH−managed REDCap. Participants receive $40 for each assessment. Participants who consent at baseline to provide a urine sample can provide a urine sample at each assessment wave. Consent at baseline carries over to all other visits, however, participants can revoke urine analysis consent without penalization. Participants receive an additional $15 compensation for each urine sample. Participants can choose if they would like the results shared with them for educational purposes.

**Table 1:** Study assessments

Assessment	Domains	Baseline	Wave 1	Wave 2	Wave 3
Demographics	Demographics	X			
HIV and HCV	HIV and HCV Status	X			X
**Substance use**					
Addiction severity index (ASI) [61]	Past 30-day substance use	X	X	X	X
Polysubstance Assessment Tool [62]	Polysubstance Use Behaviors	X	X	X	X
Solitary use	Single Item	X	X	X	X
Overdose Risk Behaviors (ORBS-2) [63]	Overdose Risk Behaviors	X	X	X	X
Overdose history	Overdose History	X	X	X	X
Risk Assessment Battery (RAB) [64]	Measure of HIV Risk Behaviors	X			X
Urine toxicology	Recent Substance Use	X	X	X	X
Criminal History	Criminal History	X			
**PTSD and mental health**					
LEC5 and Criterion A [65]	Posttraumatic Stress History	X			
PCL5 [66]	Posttraumatic Stress Symptoms	X	X	X	X
Adverse childhood experiences (ACEs) [67]	Adverse Childhood Experiences	X			
TLE-Q [68]	Traumatic life Events	X			
Negative Mood Regulation Scale [69]	Beliefs held by individuals that allow/deter them from coping with stress	X	X	X	X
Brief Symptom Inventory [70]	Brief Inventory of Psychological Symptoms	X		X	X
Difficulties in Emotional Regulation Scale (DERS) [71]	Difficulties in Emotional Regulation	X	X	X	X
Negative Mood Regulation Scale [69]	Beliefs held by individuals that allow/deter them from coping with stress	X	X	X	X
Barratt Impulsivity Scale [72]	Impulse Control	X			X
PROMIS Emotional Distress [73]	Emotional Distress	X		X	X
IIP-C [74]	Interpersonal Distress	X	X	X	X
**Other**					
Pearlin Mastery Scale [75]	Self-Perception of Life Being Under Control	X		X	X
Brief COPE [76]	Measure of Effective Coping	X		X	X
Brief Sensation Seeing Scale [77]	Assess Personality Trains of Thrill, Disinhibition, Experience Seeking, and Boredom	X			X
ISEL-12 [78]	Social Support	X		X	X
Chronic pain	Single Item Measure	X			
SF-12 + Chronic pain [79]	Physical and Mental Health Assessment	X	X	X	X
Past service utilization	Service Utilization	X			X
Technology usage	Technology Usage to Inform Future Intervention Adaptions	X			
**Intervention only**					
Working Alliance Inventory (WAI-P) [80]	Assess Relationship Between Client and Mental Healthcare Provider		X		
Client Satisfaction inventory [81]	Participant Satisfaction with Intervention		X		

Once a participant completes their final follow-up assessment or is deemed to be lost to follow-up due to inability to contact for three months, the research assistant will complete a chart abstraction to gather data on the participant’s retention in MMT, MMT dose changes, saliva drug testing results, and other toxicology, and data from admission or discharge form(s) from StartCare.

A subset of participants will be invited to a qualitative interview at the end of the study. For those assigned to intervention, the qualitative interviews will gather participant feedback about their experience with intervention content. For those assigned to TAU, qualitative interviews will gather participant feedback about their substance use and PTSD symptoms. Qualitative interviews will further inform future intervention adaptions, as needed. A total of 30 qualitative interviews will be conducted with 10 participants in the TAU group and 20 from the intervention group. Interviews are expected to take approximately 45 min.

### Primary outcomes

The primary outcomes are PSU and PTSD symptomology at the 3-month assessment. PSU will be examined as (a) number of days of use of illicit opioids and cocaine as self-reported on the ASI [55], (b) the number of substances used as reported on the ASI, in urine toxicology results, and from chart abstraction. PTSD symptomology is conceptualized as (a) PTSD symptoms using the PCL-5 [82], (b) negative affect using the Negative Mood Regulation Scale [83], and (c) interpersonal distress using the Inventory of Interpersonal Problems [84].

### Implementation outcomes

Data on the study’s feasibility and acceptability will also be assessed. Feasibility is indicated by (a) the proportion of eligible persons, (b) proportion of eligible who enroll in the study, (c) number of intervention sessions completed, and (d) interventionist perspective of feasibility. Acceptability will be assessed by (a) participant satisfaction [81] and (b) interventionist perspective of acceptability [85].

### Trial status

Recruitment is anticipated to begin in October of 2024.

## Analysis

Before analyses, randomization balance will be assessed by comparing baseline characteristics between the intervention arms. Wilcoxon’s rank sum tests for continuous variables and Fisher’s exact tests for categorical variables will be used, respectively. A two-sided *p*-value < 0.05 will be considered statistically significant. Rstudio [60] will be used to analyze all data. The primary analyses will be performed according to the Intent-To-Treat (ITT) principle with secondary analyses using a per-protocol approach given extant barriers in the patient population. After completion of the primary outcome analysis, data will be made publicly available in accordance with funder requirements.

### Analysis of implementation

The prevalence of all eligible subjects and subjects eligible to enroll in the study of all possible MMT patients approached will be reported. 95% confidence intervals based on the normal approximation will be reported. Comparisons of prevalence between intervention arms will utilize Logistic regression. Number of intervention sessions completed will also be reported. Poisson regression will be used to evaluate differences across intervention arms. Participant satisfaction will be described with a scale, ranging from 1 (Lowest satisfaction) to 7 (Highest) for 23 items [81]. Mean participant satisfaction will be computed and compared across intervention arms by using Kruskal–Wallis test. Qualitative data analysis of implementation outcomes is led by the MPIs, given their extant experience with thematic and implementation barrier analysis. Audio-recorded interviews will be transcribed by a professional transcription service. Thematic coding using a codebook created a priori and further informed during the data analysis will be used to assess outcomes noted above. Thematic coding will be guided by appropriate frameworks.

### Analysis of short-term measures

The change in PSU from baseline to wave 1, 2, and 3 across randomized groups will be used as the summary measure. A mixed-effect model with a Poisson distribution will be used to evaluate changes in PSU across baseline and waves 1, 2, and 3. The model will contain a fixed effect of the randomization group, a fixed and a random effect of the wave, and an interaction between the wave and the randomization group. All primary hypothesis tests will be two-sided using alpha 0.05 significance level, and p-values adjusted and unadjusted for multiple testing will be reported. Bonferroni correction will be used to control p-values for multiple testing. Point estimates, confidence intervals (at the 95% confidence level) will be computed. An analysis of variance (ANOVA) model will be used to test the hypothesis of no difference across randomized groups in difference in PSU. All participants will be included in analyses regardless of sex, race, or ethnicity. Subgroup analyses by sex, race, and ethnicity will be completed to examine potential sex and/or racial differences.

### Treatment of missing data

If the proportions of missing data are negligible (e.g., below 5%), a complete case analysis will be used as the primary analysis assuming missing completely at random. Otherwise, missing-at-random will be assumed (under which mixed-effect models provide unbiased results). A sensitivity analysis will be conducted imputing missing values by using Multivariate Imputation by Chained Equations.

### Trial monitoring

The study is overseen by a data safety monitoring board composed of researchers with expertise in implementation science, RCTs, the MMT treatment setting including lived expertise, and quantitative methods. Participants may withdraw consent and end their participation at any time. Individuals in the current study have concurrent substance use disorders and PTSD. Unfortunately, the likelihood of adverse events such as overdose are common in this population. However, no adverse events are expected as directly related to the study. Adverse events will be systematically collected according to IRB regulations. The data safety monitoring board will review adverse events and interim analyses at the study mid-point and make appropriate recommendations.

## Discussion

By adapting the evidence-based STAIR-NT intervention to a condensed, massed treatment model, this study explores a novel approach to improving both PTSD and substance use outcomes in this vulnerable population. The use of a massed treatment schedule addresses the practical barriers that often limit the accessibility and effectiveness of traditional, prolonged PTSD treatments. Additionally, the study's design acknowledges the complexities of treating patients with trauma histories and multiple substance use behaviors, emphasizing the need for comprehensive care that integrates both emotional regulation and narrative therapy components. This research aims to contribute to the growing body of literature advocating for trauma-informed care in the treatment of substance use disorders. Importantly, it also addresses the pressing public health issue of stimulant-opioid co-use, particularly in populations disproportionately affected by overdose fatalities and health disparities.

The THRIVE study represents a promising step toward improving outcomes for individuals with complex trauma and substance use histories. Should the results prove favorable, this approach could serve as a model for treating co-occurring PTSD and substance use in other high-risk populations.

## Data Availability

No datasets were generated or analysed during the current study.

## Abbreviations

ADAPT-ITT: Assessment, Decision, Adaptation, Production, Topical Experts, Integration, Training, Testing Framework
ANOVA: Analysis of Variance
ASI: Addiction Severity Index
BIPOC: Black, Indigenous, and People of Color
CAPI: Computer Assisted Personal Interview
DSM-V: Diagnostic and Statistical Manual of Mental Disorders, Fifth Edition
IRB: Institutional Review Board
ITT: Intent-To-Treat
LMHC: Licensed Mental Health Counselor
LMSW: Licensed Master Social Worker
MMT: Methadone Maintenance Treatment
MPI: Multiple Principal Investigator
NIDA: National Institute on Drug Abuse
NIH: National Institutes of Health
NT: Narrative therapy
NYULH: New York University Langone Health
OTP: Opioid Treatment Program
PhenX: Phenotypes and eXposures
PSU: Polysubstance Use
PTSD: Posttraumatic Stress Disorder
PCL-5: Posttraumatic Stress Disorder Checklist for DSM-5
PC-PTSD-5: Primary Care PTSD Screen for DSM-5
RCT: Randomized Controlled Trial
STAIR: Skills Training in Affective and Interpersonal Regulation
STAIR-NT: Skills Training in Affective and Interpersonal Regulation with Narrative Therapy
SUD: Substance Use Disorder
TAU: Treatments as Usual
THRIVE: Treatment for Harnessing Resiliency, Improving emotional regulation, and empowering indiViduals for a brighter future
